# Impact of breastfeeding during infancy on functional constipation at 3 years of age: the Japan Environment and Children’s Study

**DOI:** 10.1186/s13006-023-00592-y

**Published:** 2023-11-06

**Authors:** Noriko Motoki, Yuji Inaba, Hirokazu Toubou, Kohei Hasegawa, Takumi Shibazaki, Teruomi Tsukahara, Tetsuo Nomiyama, the Japan Environment and Children’s Study (JECS) Group, Michihiro Kamijima, Shin Yamazaki, Yukihiro Ohya, Reiko Kishi, Nobuo Yaegashi, Koichi Hashimoto, Chisato Mori, Shuichi Ito, Zentaro Yamagata, Hidekuni Inadera, Takeo Nakayama, Tomotaka Sobue, Masayuki Shima, Hiroshige Nakamura, Narufumi Suganuma, Koichi Kusuhara, Takahiko Katoh

**Affiliations:** 1 Center for Perinatal, Pediatric, and Environmental Epidemiology, Shinshu University School of Medicine, 3-1-1 Asahi, Matsumoto, 390-8621 Japan; 2 Department of Neurology, Nagano Children’s Hospital, Azumino, Nagano, Japan; 3 Life Science Research Center, Nagano Children’s Hospital, Azumino, Nagano, Japan; 4 Department of Preventive Medicine and Public Health, Shinshu University School of Medicine, Matsumoto, Nagano, Japan; 5 Department of Pediatrics, Shinshu University School of Medicine, Matsumoto, Nagano, Japan

**Keywords:** Functional constipation, Exclusive breastfeeding, Breastfeeding period duration, Japan, Infant, Toddler

## Abstract

**Background:**

There is a lack of large, nationwide, birth cohort studies in Japan that examine the relationships of initial feeding habits and breastfeeding period duration with offspring functional constipation at 3 years of age. This study assessed the impact of breastfeeding during infancy on early childhood functional constipation.

**Methods:**

The fixed data of 70,078 singleton births from the ongoing Japan Environment and Children’s Study cohort study that commenced in 2011 were used to identify functional constipation as estimated by Rome III at 3 years of age. The exposure variables were breastfeeding period duration until 12 months of age (never, up to 6 months, or ≥ 7 months) as well as breastfeeding status at 1 month and 6 months of age (breastfeeding exclusively, partial breastfeeding, or infant formula feeding only). Multiple logistic regression analysis was employed to search for correlations for functional constipation development with breastfeeding period duration until 12 months of age and breastfeeding status during infancy.

**Results:**

We identified 8,118 toddlers (11.6%) who met the Rome III criteria at 3 years of age. After controlling for potential covariates, a breastfeeding period duration of 7 months or more was inversely related to functional constipation development (≥ 7 months: adjusted odds ratio [OR] [95% confidence interval (CI)] 0.76 [0.65, 0.88] versus never breastfed, *P* for trend < 0.001). Other initial feeding methods were significantly related to an increased risk of functional constipation as compared with breastfeeding exclusively at 1 month of age (partial breastfeeding: adjusted OR [95% CI] 1.17 [1.11, 1.23], formula feeding only: 1.23 [1.07, 1.40]) and 6 months of age (partial breastfeeding: adjusted OR [95% CI] 1.18 [1.12, 1.24], formula feeding only: adjusted OR [95% CI] 1.42 [1.20, 1.68]).

**Conclusion:**

This large nationwide survey revealed a possible protective effect of a prolonged breastfeeding period duration and early exclusive breastfeeding in infancy on functional constipation at 3 years.

**Supplementary Information:**

The online version contains supplementary material available at 10.1186/s13006-023-00592-y.

## Background

Functional constipation is defined as chronic constipation with no identifiable underlying cause. Although functional constipation is common among children, it is also a significant reason for morbidity, accounting for up to 25% of visits to pediatric gastroenterologists [1]. The etiology of functional constipation is considered multifactorial and has not been fully clarified. Among children, dietary habits such as lower intake of fruits and vegetables, less outdoor play activity, and lower regular physical activity time were reportedly associated with functional constipation development [2–5]. Psychological stress and family problems have been identified as factors related to constipation during school age as well [5]. Nakamura et al. also postulated that delivery mode may affect gut microbiota and associate with the prevalence of functional constipation in a cohort of toddlers [6].

The health benefits of breastfeeding extend not only to short-term nutritional benefits, but also to long-term immunity and metabolism in addition to gut microbiota composition [7–9]. Furthermore, several studies have demonstrated breastfeeding as a protective factor against the development of functional constipation in infancy [10, 11]. functional constipation has been reported to be less common in breastfed infants than in infant formula-fed ones [10]. Breastfeeding status impacted stool frequency and consistency in infants aged 0–4 months, but not after weaning [11]. However, it remains uncertain whether initial breastfeeding habits and breastfeeding period duration are associated with functional constipation at 3 years of age in the late post-weaning period. Accordingly, we conducted a large birth cohort study to examine the impacts of initial feeding status and breastfeeding period until the age of 12 months on the presence of functional constipation at 3 years of age.

## Methods

### Study design and participants

The data used in this study were obtained from the Japan Environment and Children’s Study (JECS), an ongoing cohort study that began in January 2011 to identify the effect of environmental factors on children’s health.

In the JECS, pregnant women were enrolled at among 15 Regional Centers in Japan between January 2011 and March 2014. The inclusion criteria were: 1) having residence in the Study Area at the time of recruitment, 2) expected delivery after August 1, 2011, and 3) capable of comprehending the Japanese language and completing the self-administered structured questionnaire in Japanese. This study was registered in the UMIN Clinical Trials Registry (no. UMIN000030786). Details of the JECS project have been described previously [12–14]. The JECS protocol was reviewed and approved by the Ministry of the Environment’s Institutional Review Board on Epidemiological Studies (no. 100910001) as well as by the Ethics Committees of all participating institutions. The JECS was conducted in accordance with the Helsinki Declaration and other nationally valid regulations and guidelines. Written informed consent was obtained from all participants.

Most of the questionnaires during pregnancy were distributed to women attending prenatal examinations, with some sent by post. Completed questionnaires were submitted during subsequent prenatal visits or mailed. When possible, respondents giving incomplete answers were interviewed face-to-face or by telephone for missing details. The first trimester and second/third questionnaire response rates were 98.5% and 97.2%, respectively [12]. Regarding the medical record transcriptions of mothers in early pregnancy and children at birth, the response rates were both 100% [12]. After the neonatal period, surveying has continued every 6 months via self-administered questionnaires given by mothers or caregivers. If the questionnaire is not returned, reminders are sent by telephone, postcard, or text message as necessary by each Regional Center. As of September 25, 2022, the questionnaire response rates for 6-month-olds, 1-year-olds, and 3-year-olds are 94.1%, 91.4%, and 84.2%, respectively.

**Fig. 1:**
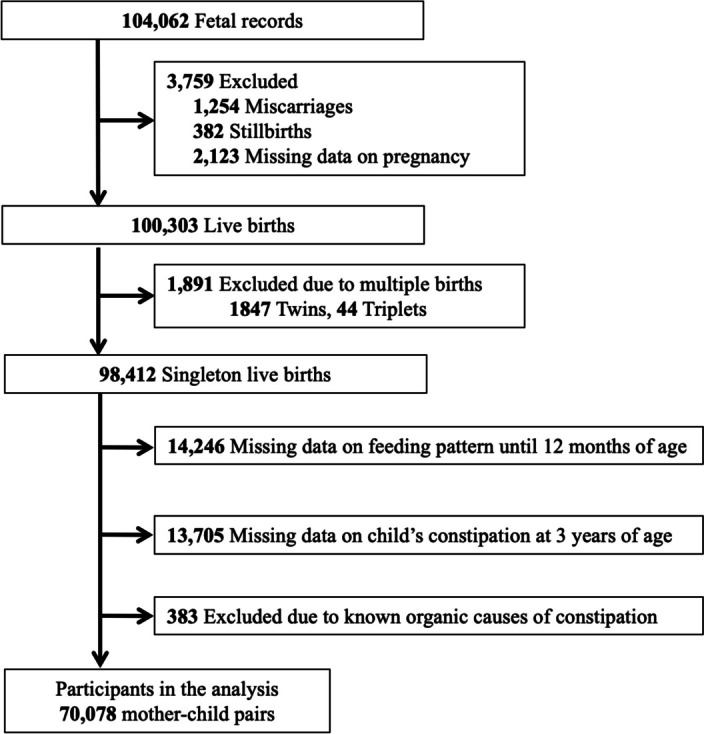
Case selection flowchart

The present study was based on the “jecs-ta-20190930” dataset released in October 2019 containing information on 98,412 singleton live births. We excluded 14,246 participants with insufficient or missing data on feeding status up to 12 months of age and 13,705 with insufficient or missing data on the child’s constipation. Participants reporting known organic causes of constipation, including Hirschsprung’s disease, spina bifida, thyroid gland insufficiency, and 21 trisomy as diagnosed by physicians, were also excluded, leaving 70,078 mother-toddler pairs for the analysis (Fig. 1).

### Data collection

#### Exposure

Data on breastfeeding status during the first month of life were derived from medical record transcripts completed by physicians, midwives/nurses, and/or Research Co-ordinators and classified into 3 categories: breastfeeding exclusively (feeding with breast milk only), partial breastfeeding (feeding with any combination of breast milk and infant formula), or formula feeding only. Information on breastfeeding status until 6 months of age and the breastfeeding period until 12 months of age were obtained from self-reported questionnaires completed by mothers, whereby all applicable month numbers could be selected for the breastfeeding period and formula feeding period.

The breastfeeding duration up to 12 months of age was defined as the period up to the last month between 1 and 12 months in which mothers checked selections for any breastfeeding, regardless of whether or not infant formula was used in combination. Mothers who checked the breastfeeding selection for the first 6 months while leaving the formula feeding box unchecked were considered as breastfeeding exclusively. Mothers who checked the formula feeding selection for the first 6 months while leaving breastfeeding unchecked were considered as formula feeding only, with all others classified as partial breastfeeding. Partial breastfeeding referred to feeding with a combination of breast milk and infant formula, regardless of which milk was predominant. Breastfeeding status in this study referred to feeding methods related to milk, and did not take into account whether or not solid food was also provided.

#### Outcomes

The main outcome of interest was the presence of functional constipation in 3-year-old toddlers of mothers whose answers met the Rome III diagnostic criteria [15, 16]. Rome III is a standard set of diagnostic criteria for childhood functional gastrointestinal disorders and requires at least 2 of the following items lasting for a minimum of 1 month for a diagnosis of functional constipation: 1) two or fewer defecations per week, 2) at least 1 episode per week of incontinence after the acquisition of toileting skills, 3) history of excessive stool retention, 4) history of painful or hard bowel movements, 5) presence of a large fecal mass in the rectum, and 6) history of large-diameter stools that may obstruct the toilet. The Japanese version of Rome III was used in this study [17].

#### Covariates

The covariates in our models were selected a priori based on previously published literature and biologic plausibility [4–6, 18–21]. We included the following covariates for mothers and their partners: maternal age at delivery (< 35 or ≥ 35 years), pre-pregnancy body mass index (BMI) (< 18.5, 18.5–24.9, 25.0–29.9, or ≥ 30.0 kg/m^2^), parity (primipara or multipara), cesarean birth (yes or no), maternal highest education age (< 16, 16 to < 19, or ≥ 19 years), marital status (married, single, divorced, or widowed), and annual household income (< 2 million, 2 million to < 4 million, 4 million to < 6 million, 6 million to < 8 million, or ≥ 8 million Japanese yen). As covariates for children, we included gender (male or female), gestational age (< 37, 37–41, or ≥ 42 weeks), birth weight (< 1,500, 1,500–2,499, 2,500–3,999, or ≥ 4,000 g), and started solid baby food by 6 months of age (yes or no). Pre-pregnancy body BMI to evaluate body weight status for mothers was calculated according to World Health Organization Standards as body weight (kg)/height (m)^2^.

### Statistical analysis

Distribution normality was confirmed by the Kolmogorov–Smirnov test. Data are expressed as the mean ± standard deviation or the median (interquartile range) depending on whether they are normally distributed or not, respectively. The outcome of interest was functional constipation defined as any toddler satisfying the Rome III diagnostic criteria as described by the mother. The exposure variables were breastfeeding period until 12 months of age (never [reference], up to 6 months, or ≥ 7 months), breastfeeding status at 1 month of age (breastfeeding exclusively [reference], partial breastfeeding, or formula feeding only), and breastfeeding exclusively at 6 months of age (breastfeeding exclusively [reference], partial breastfeeding, or formula feeding only). We adopted logistic regression models to calculate crude and adjusted odds ratios (ORs) and their 95% confidence intervals (CIs). The forced entry method was used to enter covariates into multivariable analysis models. Model 1 was adjusted for demographic and prenatal covariates, including maternal age at delivery, pre-pregnancy BMI, maternal highest level of education, annual household income, marital status, maternal gestational weight gain, and parity. Model 2 was adjusted for the covariates in model 1 in addition to covariates after birth, including cesarean section at delivery, gender, birth weight, and started solid baby food by 6 months of age. The JECS protocol prohibits sharing the ORs of covariates to prevent double publications because other JECS studies use covariates as outcomes in parallel. Missing data on covariates were excluded in the logistic regression models. Spearman’s rank correlation coefficient was used to check for multicollinearity of covariates. Hosmer–Lemeshow testing was employed to assess the goodness-of-fit of the models. All statistical analyses were performed using SPSS statistical software version 29 (SPSS Inc., Chicago, Illinois).

## Results

A total of 70,078 mothers with singleton live births were available for analysis (Fig. 1). Table 1 summarizes the participants’ characteristics in terms of breastfeeding period duration. Among the participants, 1,623 (2.3%) never breastfed, 14,174 (20.2%) breastfed up to 6 months, and 54,281 (77.5%) breastfed for ≥ 7 months. We observed significant differences among the groups regarding maternal age, pre-pregnancy BMI, gestational weight gain, annual household income, marital status, parity, cesarean section, gestational age, birth weight, and started solid baby food by 6 months of age (all *P* < 0.05).

**Table 1:** Characteristics of participants according to breastfeeding period duration (*n* = 70,078)

Variable	Breastfeeding period duration		
	Never	Up to 6 months	≥ 7 months
	*n* = 1,623	*n* = 14,174	*n* = 54,281
Maternal age at delivery (years)	31 (27, 35)	31 (27, 35)	31 (28, 35)
Maternal age group, n (%)			
< 35 years	1,132 (69.7)	10,376 (73.2)	39,205 (72.2)
≥ 35 years	491 (30.3)	3,797 (26.8)	15,076 (27.8)
Pre-pregnancy BMI (kg/m^2^)	21.2 (19.3, 24.0)	20.8 (19.1, 23.2)	20.4 (19.1, 22.2)
Pre-pregnancy BMI group, n (%)			
Underweight (BMI < 18.5)	248 (15.3)	2,246 (15.9)	8,865 (16.3)
Normal weight (BMI 18.5–24.9)	1,055 (65.1)	9,843 (69.5)	40,887 (75.4)
Overweight (BMI 25.0–29.9)	242 (14.9)	1,531 (10.8)	3,604 (6.6)
Obese (BMI ≥ 30.0)	75 (4.6)	550 (3.9)	891 (1.6)
Gestational weight gain (kg)	10.2 (7.6, 12.8)	10.3 (7.8, 13.0)	10.1 (8.0, 12.4)
Highest age of maternal education, n (%)			
< 16 years	178 (11.1)	918 (6.6)	1,380 (2.6)
16 to < 19 years	759 (47.5)	5,907 (42.2)	13,986 (26.0)
≥ 19 years	662 (41.4)	7,181 (51.3)	38,479 (71.5)
Annual household income^a^, n (%)			
< 2,000,000 JPY	164 (11.2)	1,067 (8.2)	1,983 (3.9)
2,000,000–3,999,999 JPY	592 (40.6)	5,112 (39.5)	16,377 (32.1)
4,000,000–5,999,999 JPY	451 (30.9)	4,019 (31.0)	17,580 (34.5)
6,000,000–7,999,999 JPY	154 (10.6)	1,654 (12.8)	9,014 (17.7)
≥ 8,000,000 JPY	97 (6.7)	1,096 (8.5)	6,055 (11.9)
Marital status, n (%)			
Married	1,507 (94.0)	13,188 (94.1)	52,227 (96.9)
Single	73 (4.6)	659 (4.7)	1,431 (2.7)
Divorced	23 (1.4)	172 (1.2)	226 (0.4)
Widowed	0 (0.0)	2 (0.0)	10 (0.0)
Parity, n (%)			
Primiparous	615 (38.7)	5,670 (40.9)	22,021 (41.6)
Multiparous	975 (61.3)	8,187 (59.1)	30,959 (58.4)
Cesarean section at delivery, n (%)			
No	1,214 (75.0)	11,086 (78.3)	44,942 (83.0)
Yes	405 (25.0)	3,066 (21.7)	9,216 (17.0)
Offspring gender (male), n (%)	824 (50.8)	7,256 (51.2)	27,587 (50.8)
Gestational age (weeks)	39 (38, 40)	39 (38, 40)	39 (38, 40)
Gestational age group, n (%)			
< 37 weeks	120 (7.4)	869 (6.1)	2,017 (3.7)
37–41 weeks	1,497 (92.4)	13,263 (93.6)	52,115 (96.0)
≥ 42 weeks	3 (0.2)	37 (0.3)	128 (0.2)
Birth weight (g)	2,994 (2,720, 3,269)	3,000 (2,746, 3,268)	3,040 (2,798, 3,286)
Birth weight group, n (%)			
< 1,500	12 (0.7)	100 (0.7)	171 (0.3)
1,500–2,499 g	183 (11.3)	1,320 (9.3)	3,545 (6.5)
2,500–3,999 g	1,411 (87.1)	12,605 (89.0)	50,095 (92.3)
≥ 4000 g	14 (0.9)	142 (1.0)	439 (0.8)
Started solid baby food by 6 months of age, n (%)			
No	372 (23.0)	2,971 (21.0)	15,255 (28.2)
Yes	1,246 (77.0)	11,172 (79.0)	38,925 (71.8)

Continuous variables are expressed as the mean ± standard deviation or the median (interquartile range)

Breastfeeding duration up to 12 months of age was defined as the period up to the last month between 1 and 12 months in which mothers checked selections for any breastfeeding, regardless of whether or not infant formula was used in combination

Data were missing on maternal age (*n* = 1), pre-pregnancy BMI (*n* = 41), body weight gain during pregnancy (*n* = 637), maternal education level (*n* = 628), household income (*n* = 4,663), marital status (*n* = 560), parity (*n* = 1,651), mode of delivery (*n* = 149), birth weight (*n* = 41), gestational age (*n* = 29), and started solid baby food by 6 months of age (*n* = 137)

BMI, body mass index; JPY, Japanese yen

^a^The average (median) annual Japanese household income in 2018 was 5,523,000 JPY (4,370,000 JPY). The currency exchange rates on January 25, 2023, were: 1 USD = 130 JPY and 1 EUR = 142 JPY

We identified 8,118 toddlers (11.6%) who met the Rome III criteria at 3 years of age (Tables 2 and 3). In logistic regression analysis after adjustment for covariates, a breastfeeding period duration of ≥ 7 months was inversely related to the development of functional constipation as compared with never breastfed (adjusted OR [95% CI] 0.75 [0.64, 0.87] in model 1, 0.76 [0.65, 0.88] in model 2, both *P* for trend < 0.001) (Table 2). Other early feeding methods were significantly related to an increased risk of functional constipation as compared with breastfeeding exclusively at 1 month of age (partial breastfeeding: adjusted OR [95% CI] 1.17 [1.11, 1.23], formula feeding: 1.23 [1.07, 1.40] in model 2) and 6 months of age (partial breastfeeding: adjusted OR [95% CI] 1.18 [1.12, 1.24], formula feeding: 1.42 [1.20, 1.68] in model 2) (Table 3).

**Table 2:** Odds ratio and 95% confidence interval of associations between breastfeeding duration up to 12 months of age and functional constipation at 3 years of age

	Without functional constipation, n	With functional constipation, n (%)	Model 1	Model 2
	*n* = 61,960	*n* = 8,118 (11.6)	adjusted OR (95%CI)	adjusted OR (95% CI)
Breastfeeding duration				
Never (reference)	1,378	245 (15.1)	1.00	1.00
Up to 6 months	12,242	1,932 (13.6)	0.92 (0.78, 1.07)	0.92 (0.79, 1.08)
≥ 7 months	48,340	5,941 (10.9)	**0.75 (0.64, 0.87)**	**0.76 (0.65, 0.88)**
* P* for trend			**< 0.001**	**< 0.001**

Breastfeeding duration up to 12 months of age was defined as the period up to the last month between 1 and 12 months in which mothers checked selections for any breastfeeding, regardless of whether or not infant formula was used in combination

Model 1 was adjusted for demographic and prenatal covariates, including maternal age at delivery, pre-pregnancy body mass index, maternal gestational weight gain, maternal highest age of education, annual household income during pregnancy, marital status, and parity

Model 2 was adjusted for the covariates in model 1 in addition to covariates after birth, including cesarean section at delivery, birth weight, gender, and started solid baby food by 6 months of age

*OR* odds ratio, *CI* confidence interval

**Table 3:** Odds ratio and 95% confidence interval of associations between exclusive breastfeeding and functional constipation at 3 years of age

	Without functional constipation, n	With functional constipation, n (%)	Model 1	Model 2
	*n* = 61,960	*n* = 8,118 (11.6)	adjusted OR (95%CI)	adjusted OR (95% CI)
Breastfeeding status at 1 month of age				
Breastfeeding exclusively (reference)	35,192	4,173 (10.6)	1.00	1.00
Partial breastfeeding	24,767	3,631 (12.8)	**1.17 (1.11, 1.23)**	**1.17 (1.11, 1.23)**
Formula feeding only	2,001	314 (13.6)	**1.23 (1.08, 1.41)**	**1.23 (1.07, 1.40)**
Breastfeeding status at 6 months of age				
Breastfeeding exclusively (reference)	25,603	2,921 (10.2)	1.00	1.00
Partial breastfeeding	35,190	4,985 (12.4)	**1.18 (1.12, 1.24)**	**1.18 (1.12, 1.24)**
Formula feeding only	1,167	212 (15.4)	**1.42 (1.21, 1.68)**	**1.42 (1.20, 1.68)**

Breast feeding exclusively was defined as feeding with breast milk only. Formula feeding only was defined as feeding with infant formula only. Partial breastfeeding referred to feeding with any combination of breast milk and infant formula. Breastfeeding status referred to feeding methods related to milk, and did not take into account whether or not solid food was also provided

Model 1 was adjusted for demographic and prenatal covariates, including maternal age at delivery, pre-pregnancy body mass index, maternal gestational weight gain, maternal highest age of education, annual household income during pregnancy, marital status, and parity

Model 2 was adjusted for the covariates in model 1 in addition to covariates after birth, including cesarean section at delivery, birth weight, gender, and started solid baby food by 6 months of age

*OR* odds ratio, *CI* confidence interval

Lastly, we analyzed the sociodemographic characteristics of 98,412 participants by including the 28,334 participants originally excluded from the final analysis (Additional file 1). We observed significant differences for the proportion of maternal age, pre-pregnancy BMI, highest age of maternal education, annual household income, marital status, and parity, as well as breastfeeding status during infancy, between the analyzed and excluded participants. Notably, missing data among the excluded participants were predominated by highest maternal education, and annual household income.

## Discussion

We herein describe the first large-scale nationwide birth cohort study in Japan examining the relationship of breastfeeding habits with offspring functional constipation at 3 years of age. Our results indicate that a longer breastfeeding period duration prior to 12 months of age and breastfeeding exclusively during infancy may significantly decrease the risk of functional constipation.

Constipation is likely to occur in childhood under several reported conditions: 1) weaning period during the transition from breast milk to artificial milk or the start of baby food, 2) toilet training in toddlers, and 3) school-childhood when children begin going to school but avoid defecation there [22]. The peak age of functional constipation onset is considered to be while toilet training, i.e., between 2 and 4 years of age [22]. In the present study, the prevalence rate of functional constipation in 3-year-old toddlers was 11%, which was higher than the 9.4% in a US study [23], 9.7% in a European study [21], and 5.6–8% in other Asian countries [4, 24, 25]. Differences in genetic background, dietary habits, and toilet training methods, as well as the starting time of daycare, nursery school, and kindergarten are considered to account for such discrepancies.

Dietary habits, fluid intake, exercise, socioeconomic variables, and psychological factors all play prominent roles in the risk of functional constipation among young children [3–5]. Nakamura et al. also revealed delivery mode to be associated with functional constipation prevalence among toddlers; at 3 years of age, children born by cesarean delivery had a significantly higher risk of suffering functional constipation than those born by vaginal delivery [6]. Differences in delivery modes have been linked to variability in intestinal microbiota composition, which may influence the development of functional constipation in both infancy and early childhood [26, 27]. In a prospective study, the gut microbiota of children with functional constipation diagnosed by Rome III criteria could be clearly discriminated from that of healthy controls [18]. Supporting this, several reports evaluating the gut microbiota of children with constipation revealed lower levels *Lactobacillus* species and higher amounts of *Bacteroides* species and *Bifidobacterium longum* than in controls [18, 28–30]. Such alterations in intestinal microbiota could factor in pediatric functional constipation occurrence.

Breastfeeding is among the principal factors that support healthy physical and neurological development and is actively promoted by the World Health Organization [7]. The American Academy of Pediatrics recommends breastfeeding exclusively for roughly the first 6 months, followed next by nutritionally adequate and safe complementary foods while continuing breastfeeding for 1 year or longer as mutually desired by the mother and infant [8]. The benefits of breastfeeding also include protection against respiratory tract and gastrointestinal infections, allergies, and obesity, with enhanced neurodevelopmental outcomes [8]. Although breastfeeding period duration and breastfeeding initiation were reportedly associated with functional constipation in infants of less than 4 months of age, there is controversy in the post-weaning period [3, 11, 31–33]. Our data indicate that such effects may persist until as late as the age of 3 years after adjusting for covariates including socioeconomic parameters and perinatal information.

It is uncertain why early breastfeeding initiation and sustained period duration are associated with reduced functional constipation risk during early childhood, as indicated in this study. One mechanism may be the influence of the aforementioned gut microbiota. Microbiome profiles during the neonatal and infancy periods are strongly affected by the mode of delivery and feeding [34]. Children with functional constipation are more likely to have a history of cesarean delivery and a shorter period of breastfeeding than those with no such history [28]. Breastfeeding, either exclusively or partially, was more significantly associated with microbiome composition than was birth mode [34]. Together with the fact that Zhong et al. demonstrated breastfeeding period duration in early life as related to gut microbiota make-up even in school-age children [9], our findings suggested that early gut microbiome disturbances influenced by a shorter breastfeeding period duration might increase the risk of developing functional constipation later in childhood.

This study had several limitations. First, the data on as measured by Rome III were collected from caregiver self-reported questionnaires and therefore considered subjective. Second, as the findings on functional constipation were evaluated at 3 years of age, children with functional constipation onset and improvement before or later than that age were not considered. Third, the data on feeding method until 12 months of age were also considered subjective as they were obtained from self-reported questionnaires completed by mothers. Moreover, this study’s definition of partial breastfeeding included a combination of breast milk and infant formula; however, the cut-off points for being predominantly formula-fed or breastfed were not assessed. Fourth, the large attrition rate of participants lacking information on feeding by age 12 months as well as those not completing Rome III may have constituted selection bias. Also shown in Additional file 1, there were significant differences in several sociodemographic parameters and breastfeeding information between the analyzed and excluded participants. Therefore, we cannot conclusively rule out the possibility of under-reporting the incidence of functional constipation. Finally, information on dietary habits including fruit and vegetable intake, water intake, and fast food consumption, all of which play a considerable role in the development of constipation, in addition to the genetic background of constipation, were unavailable and could not be included as covariates.

## Conclusions

This is the first study employing a large dataset from a Japanese nationwide birth cohort study to analyze the impact of breastfeeding in infants on the presence of functional constipation at 3 years of age. After controlling for potential confounders, a longer breastfeeding period duration and earlier exclusive breastfeeding were identified to possibly decrease the risk of functional constipation. Such findings may constitute supportive evidence on recommending breastfeeding exclusively in early life and continued breastfeeding after weaning.

### Supplementary Information

**Additional file 1. **Sociodemographic characteristics and breastfeeding information between analyzed and excluded participants (*n* = 98,412).

## Data Availability

Data are unsuitable for public deposition due to ethical restrictions and legal framework of Japan. It is prohibited by the Act on the Protection of Personal Information (Act No. 57 of 30 May 2003, amendment on 9 September 2015) to publicly deposit the data containing personal information. Ethical Guidelines for Medical and Health Research Involving Human Subjects enforced by the Japan Ministry of Education, Culture, Sports, Science and Technology and the Ministry of Health, Labour and Welfare also restricts the open sharing of the epidemiologic data. All inquiries about access to data should be sent to: jecs-en@nies.go.jp. The person responsible for handling enquiries sent to this e-mail address is Dr Shoji F. Nakayama, JECS Programme Office, National Institute for Environmental Studies.

## Abbreviations

BMI: Body mass index
CI: Confidence interval
JECS: The Japan Environment and Children’s Study
OR: Odds ratio
