# Supplementary material for: Impact of breastfeeding during infancy on functional constipation at 3 years of age: the Japan Environment and Children’s Study
Source: Int Breastfeed J. 2023 Nov 6;18:57. doi: 10.1186/s13006-023-00592-y (PMC10626743; doi:10.1186/s13006-023-00592-y)
Supplement: Supplementary file 1 — Additional file 1. Sociodemographic characteristics and breastfeeding information between analyzed and excluded participants (n = 98,412). [file 13006_2023_592_MOESM1_ESM.docx]

| **Additional file 1. Sociodemographic characteristics and breastfeeding information between analyzed and excluded participants (n = 98,412)** | | | | |
| --- | --- | --- | --- | --- |
| Variable | | Analyzed participants | Excluded participants | *P*-value |
|  |  | n = 70,078 | n = 28,334 |  |
| Maternal age group, n (%) | |  |  | < 0.001 |
|  | < 35 years | 50,713 (72.4) | 21,155 (74.7) |  |
|  | ≥ 35 years | 19,364 (27.6) | 7,169 (25.3) |  |
|  | Missing | 1 (0.0) | 10 (0.0) |  |
| Pre-pregnancy BMI group, n (%) | |  |  | < 0.001 |
|  | Underweight (BMI < 18.5 kg/m^2^) | 11,359 (16.2) | 4,554 (16.1) |  |
|  | Normal weight (BMI 18.5–24.9 kg/m^2^) | 51,785 (73.9) | 20,036 (70.7) |  |
|  | Overweight (BMI 25.0–29.9 kg/m^2^) | 5,377 (7.7) | 2,690 (9.5) |  |
|  | Obese (BMI ≥ 30.0 kg/m^2^) | 1,516 (2.2) | 966 (3.4) |  |
|  | Missing | 41 (0.1) | 88 (0.3) |  |
| Highest age of maternal education, n (%) | |  |  | < 0.001 |
|  | < 16 years | 2,476 (3.5) | 2,187 (7.7) |  |
|  | 16 to < 19 years | 20,652 (29.5) | 9,610 (33.9) |  |
|  | ≥ 19 years | 46,322 (66.1) | 14,901 (52.6) |  |
|  | Missing | 628 (0.9) | 1,636 (5.8) |  |
| Annual household income*, n (%) | |  |  | < 0.001 |
|  | < 2,000,000 JPY | 2,991 (4.3) | 582 (2.1) |  |
|  | 2,000,000–3,999,999 JPY | 19,015 (27.1) | 2,918 (10.3) |  |
|  | 4,000,000–5,999,999 JPY | 23,726 (33.9) | 3,511 (12.4) |  |
|  | 6,000,000–7,999,999 JPY | 12,358 (17.6) | 1,812 (6.4) |  |
|  | ≥ 8,000,000 JPY | 8,808 (12.6) | 1,335 (4.7) |  |
|  | Missing | 3,180 (4.5) | 18,176 (64.1) |  |
| Marital status, n (%) | |  |  | < 0.001 |
|  | Married | 66,922 (95.5) | 25,596 (90.3) |  |
|  | Single | 2,163 (3.1) | 1,226 (4.3) |  |
|  | Divorced | 421 (0.6) | 385 (1.4) |  |
|  | Widowed | 12 (0.0) | 3 (0.0) |  |
|  | Missing | 560 (0.8) | 1,124 (4.0) |  |
| Parity, n (%) | |  |  | < 0.001 |
|  | Primiparous | 28,306 (40.4) | 10,347 (36.5) |  |
|  | Multiparous | 40,121 (57.3) | 17,249 (60.9) |  |
|  | Missing | 1,651 (2.4) | 738 (2.6) |  |
| Breastfeeding duration, n (%) | |  |  | < 0.001 |
|  | Never | 1,623 (2.3) | 829 (2.9) |  |
|  | Up to 6 months | 14,174 (20.2) | 4,895 (17.3) |  |
|  | 7–11 months | 14,684 (21.0) | 3,460 (12.2) |  |
|  | 12 months | 39,597 (56.5) | 9,576 (33.8) |  |
|  | Missing | 0 (0) | 9,574 (33.8) |  |
| Breastfeeding initiation at 1 month of age | |  |  | < 0.001 |
|  | Breastfeeding exclusively | 39,365 (56.2) | 12,216 (44.5) |  |
|  | Partial breastfeeding | 28,398 (40.5) | 10,641 (38.8) |  |
|  | Formula feeding only | 2,315 (3.3) | 1,637 (6.0) |  |
|  | Missing | 0 (0) | 2,945 (10.7) |  |
| Breastfeeding at 6 months of age | |  |  | < 0.001 |
|  | Breastfeeding exclusively | 28,524 (40.7) | 6,723 (23.7) |  |
|  | Partial breastfeeding  Formula feeding only  Missing | 40,175 (57.3)  1,379 (2.0)  0 (0) | 11,234 (39.6)  670 (2.4)  9,707 (34.3) |  |

BMI, body mass index; JPY, Japanese yen.

^*^ The average (median) annual Japanese household income in 2018 was 5,523,000 JPY (4,370,000 JPY). The currency exchange rates on January 25, 2023, were: 1 USD = 130 JPY and 1 EUR = 142 JPY.

Breastfeeding duration up to 12 months of age was defined as the period up to the last month between 1 and 12 months in which mothers checked selections for any breastfeeding, regardless of whether or not infant formula was used in combination.

Breast feeding exclusively was defined as feeding with breast milk only. Formula feeding only was defined as feeding with infant formula only. Partial breastfeeding referred to feeding with any combination of breast milk and infant formula. Breastfeeding status referred to feeding methods related to milk, and did not take into account whether or not solid food was also provided.
